# Supplementary material for: Severe COVID-19 patients have impaired plasmacytoid dendritic cell-mediated control of SARS-CoV-2
Source: Nat Commun. 2023 Feb 8;14:694. doi: 10.1038/s41467-023-36140-9 (PMC9907212; doi:10.1038/s41467-023-36140-9)
Supplement: Supplementary file 4 — Source Data [file 41467_2023_36140_MOESM4_ESM.zip › Source data/Venet Fig 2b_mDC2s_def/2022-04-28_Thu_17-03-27_GradientBoostClassifier_1/Confusion_matrix_pondarated Downsampling 9.pdf]

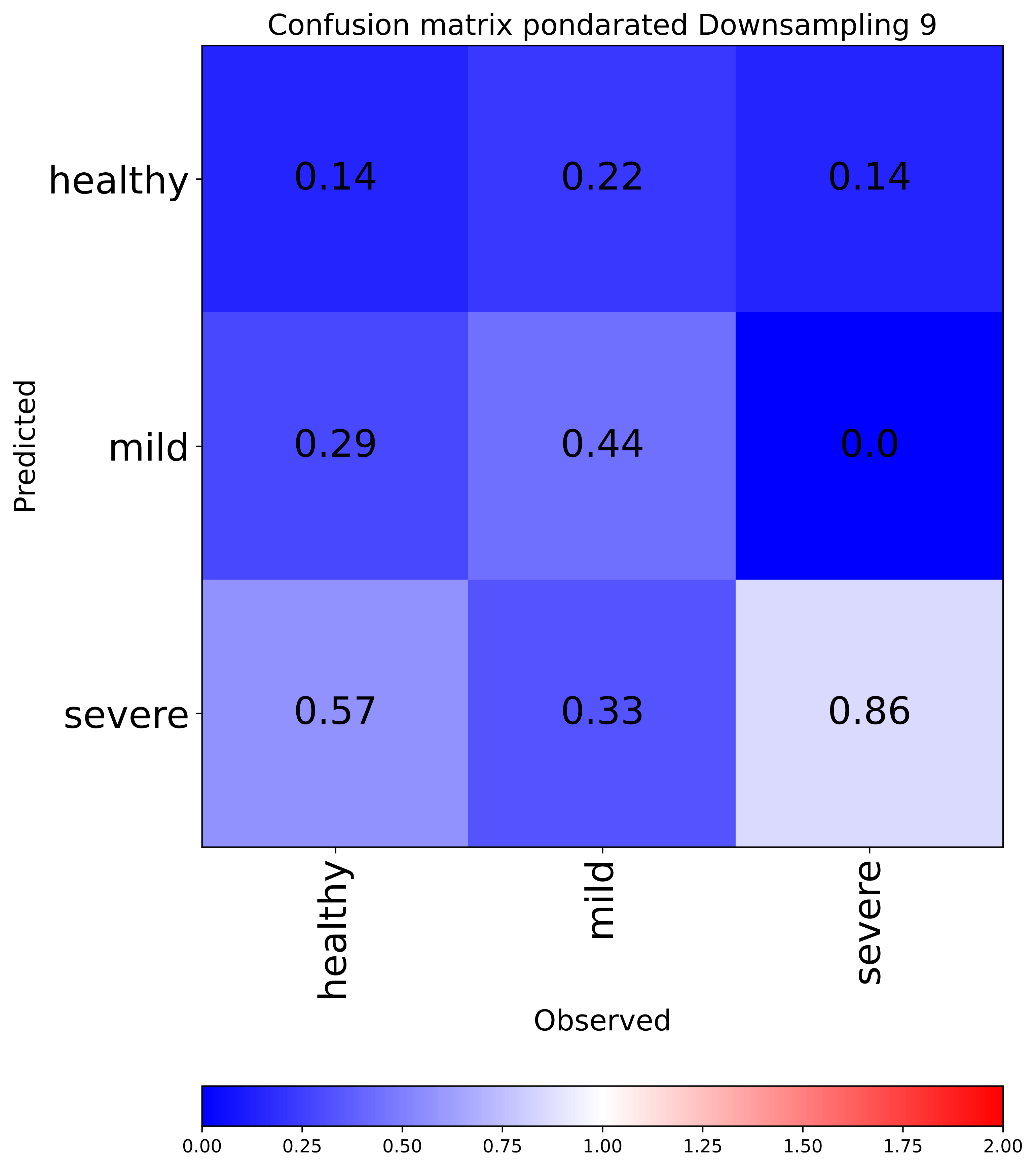
Confusion matrix pondarated Downsampling 9
Predicted
healthy
mild
severe
0.14
0.22
0.14
0.29
0.44
0.0
0.57
0.33
0.86
healthy
mild
severe
Observed
0.00
0.25
0.50
0.75
1.00
1.25
1.50
1.75
2.00
